# Relationship between regulatory pattern of gene expression level and gene function

**DOI:** 10.1371/journal.pone.0177430

**Published:** 2017-05-11

**Authors:** Masayo Inoue, Katsuhisa Horimoto

**Affiliations:** Molecular Profiling Research Center for Drug Discovery, National Institute of Advanced Industrial Science and Technology (AIST), 2-4-7 Aomi, Koto-ku, Tokyo 135-0064, Japan; Jawaharlal Nehru University, INDIA

## Abstract

Regulation of gene expression levels is essential for all living systems and transcription factors (TFs) are the main regulators of gene expression through their ability to repress or induce transcription. A balance between synthesis and degradation rates controls gene expression levels. To determine which rate is dominant, we analyzed the correlation between expression levels of a TF and its regulated gene based on a mathematical model. We selected about 280,000 expression patterns of 355 TFs and 647 regulated genes using DNA microarray data from the Gene Expression Omnibus (GEO) data repository. Based on our model, correlation between the expressions of TF–regulated gene pairs corresponds to tuning of the synthesis rate, whereas no correlation indicates excessive synthesis and requires tuning of the degradation rate. The gene expression relationships between TF–regulated gene pairs were classified into four types that correspond to different gene regulatory mechanisms. It was surprising that fewer than 20% of these genes were governed by the familiar regulatory mechanism, i.e., through the synthesis rate. Moreover, we performed pathway analysis and found that each classification type corresponded to distinct gene functions: cellular regulation pathways were dominant in the type with synthesis rate regulation and terms associated with diseases such as cancer, Parkinson’s disease, and Alzheimer’s disease were dominant in the type with degradation rate regulation. Interestingly, these diseases are caused by the accumulation of proteins. These results indicated that gene expression is regulated structurally, not arbitrarily, according to the gene function. This funding is indicative of a systematic control of transcription processes at the whole-cell level.

## Introduction

Gene expression is an essential process for all living systems [1, 2]. In general, expression levels are controlled via the balance between the synthesis rate and the degradation rate. When the synthesis rate is dominant, the expression level of a regulated gene is controlled by the expression level of a transcription factor (TF). In each transcription process, a TF induces or represses the expression of the gene alone or with the help of other proteins constituting a complex [3–5]. More than 2,000 TFs are thought to be encoded in the human genome [6, 7] and the expression levels of many genes are actually controlled through the synthesis rate. In contrast, some genes are not regulated by the synthesis rate, but by TFs that simply set the on/off state of the synthesis process and are not responsible for the synthesis rate [8, 9]. In such cases, the expression level is regulated via the degradation process; i.e. the degradation rate is dominant for the control of the expression level. Thus, the transcription of some genes is regulated by the synthesis rate, and the transcription of other genes is based on on/off regulation. However, which rate is dominant for each gene is still unclear.

The regulatory mechanisms of some genes have been studied intensively, but a comprehensive study is still difficult from a technological standpoint. Recent advances in protein quantification technologies have enabled draft maps of the human proteome to be analyzed [10, 11]; however, no high-throughput technology is currently available for analyzing the abundance of proteins at the whole-cell level. It has been widely reported that alterations in protein abundance are strongly associated with changes in mRNA expression levels [10, 12–14]. Based on this reported relationship, we used available mRNA data [15–18] to obtain a perspective view for the regulatory mechanisms of each gene at the whole-cell level.

Here, our objective is to determine which rate is dominant, the synthesis rate or the degradation rate, in the control of each gene expression level. Based on a simple mathematical model, the expression levels of a TF and the regulated gene show a correlation when the synthesis rate is dominant, but no such correlation is shown when the degradation rate is dominant. We studied this correlation by constructing approximately 280,000 scatter diagrams of “TF–regulated gene” pairs. All the scatter diagrams were classified into four types depending on the regulatory mechanisms. We also characterized each type in terms of gene function and found that the regulatory mechanisms were assigned systematically (not arbitrarily), according to the gene functions. This result illustrates that the regulatory mechanisms of gene expression levels correspond to gene function at the whole-cell level.

## Results

### Four types in scatter diagrams of TFs and regulated genes

We constructed about 280,000 scatter diagrams of expression levels of TFs and their regulated genes using DNA microarray data from the Gene Expression Omnibus (GEO; http://www.ncbi.nlm.nih.gov/geo/) at the NCBI [19]. We selected 135 series of GEO DataSets (GDS) for *Homo sapiens* that were composed of more than 50 GEO samples. The list of the selected GDS records is in S1 Table. For details of the data preparation, see Methods. To match TFs to regulated genes, we used the TRANSFAC database provided by BioBase and selected 2,073 TF–regulated gene pairs from among 355 TFs and 647 regulated genes. Note that we analyzed TF–regulated gene pairs only with regulations are already confirmed.

**Fig 1:**
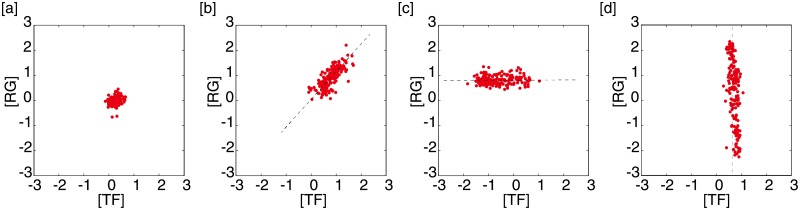
The four typical types of the scatter diagrams for TF–regulated gene pairs. The expression level of a transcription factor (TF; X-axis) and the regulated gene (Y-axis) are plotted. Data from GDS1962 were used. One point represents one sample in the GDS, namely, 180 points are shown in each diagram. (**a**) The no-change type; both a TF (*RELA*) and the regulated gene (*IKBKE*) are expressed at constant levels with small fluctuations. (**b**) The correlation type: a strong correlation between a TF (*STAT1*) and its regulated gene (*PSMB9*). (**c**) The horizontal-distribution type: a regulated gene (*CTNNB1*) shows a constant expression level regardless of changes in the TF (*NKX2-5*) expression level. (**d**) The vertical-distribution type: a regulated gene (*CCK*) undergoes changes in the expression level even at a constant expression level of the TF (*CREB1*).

We identified four typical types of scatter diagrams depending on the regulatory mechanisms between the TF–regulated gene pairs (Fig 1): constant expression levels for both a TF and the regulated gene, albeit with small fluctuations (no-change type); correlation between expression levels of a TF and the regulated gene (correlation type); no correlation between expression levels of a TF and the regulated gene because the gene has a constant expression level (horizontal-distribution type); and no correlation between expression levels of a TF and the regulated gene because the TF has a constant expression level (vertical-distribution type). We analyzed 2,073 (pairs) ×135 (GDS) to give approximately 280,000 scatter diagrams, and all the diagrams could be classified into these four types.

### Classification of TF–regulated gene relationship in four correlation types

**Fig 2:**
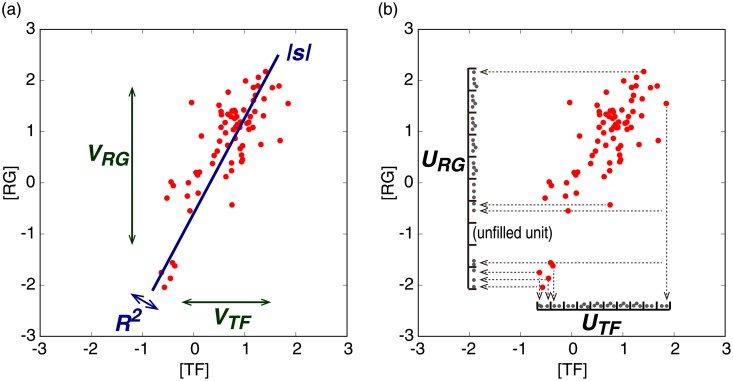
The six classification indicators used to classify the scatter diagrams. (**a**) An absolute value of slope (|*s*|) and a coefficient of determination (*R*^2^) defined from a least squares approximation, and variance in TF distribution (*V*_*TF*_) or regulated gene distribution (*V*_*RG*_) representing characteristic ranges for data distributions. (**b**) The uniformity count for a TF (*U*_*TF*_) or its regulated gene (*U*_*RG*_) defined as the number of filled units among 10 units dividing the area between a maximum and a minimum value, respectively. A unit is filled if there is at least one data point in it and it is unfilled if there are no data points.

We studied how the four classification types are implemented. We also characterized each scatter diagram with six indicators to define the classification criteria. Four of them are standard variables: absolute value of slope (|*s*|) and coefficient of determination (*R*^2^) from a least squares approximation, and variance in TF distribution (*V*_*TF*_) or regulated gene distribution (*V*_*RG*_). The other two parameters, the uniformity count for a TF (*U*_*TF*_) or its regulated gene (*U*_*RG*_), were introduced to distinguish a uniform distribution from the no-change type with a few outliers. The uniformity count is defined as the number of filled units when the area between a maximum and a minimum value is divided into 10 units. These indicators are shown schematically in Fig 2.

#### The no-change type

The no-change type (Fig 1a) is trivial and is the type that occurs most frequently because this relationship exists when there is no need to change the expression levels of both a TF and the regulated gene under the experimental conditions. Environmental changes are known to change the expression levels of some relevant genes, while the expression levels of many other genes are unchanged (thereby contributing to homeostasis, an essential attribute for all living organisms). In addition, the experimental conditions for each GDS differed and only some specific genes were affected. Thus, the observation that many genes have constant expression levels is only natural.

#### The correlation and horizontal-distribution types

The mechanisms of the correlation type (Fig 1b) and the horizontal-distribution type (Fig 1c) can be described by a simple mathematical model for a transcription process (see Methods for details). Suppose a TF molecule stochastically binds to or dissociates from a promoter sequence, and the regulated gene is transcribed only when the TF binds to the sequence. Assuming an equilibrium state, the mRNA level of the regulated gene in a steady state ([*RG*]*) can be written as a function of the expression level of the TF in the steady state ([*TF*]*) as,

$$\begin{array}{c} {\left[RG\right]}^{*}=\frac{1}{\gamma }\frac{{\left[TF\right]}^{*}/K}{1+{\left[TF\right]}^{*}/K}. \end{array}$$ (1)

**Fig 3:**
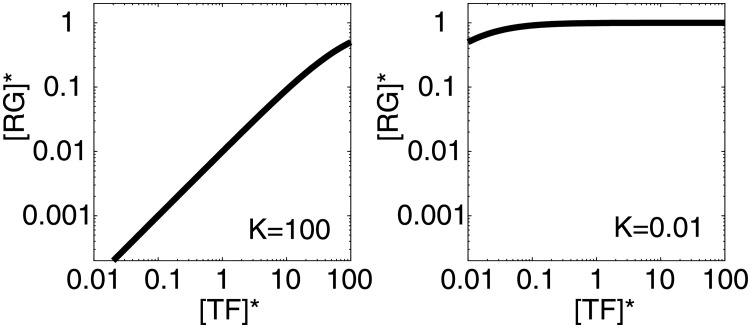
The correlation and horizontal-distribution relationships between TF–regulated gene pairs. Typical examples of the relation between a transcription factor ([*TF*]*: the X-axis) and the regulated gene ([*RG*]*: the Y-axis) according to Eq (1). Both axes have a logarithmic scale. [*RG*]* changes as a function of [*TF*]* when *K* ≫ 1 (left). [*RG*]* maintains a constant expression level when *K* ≪ 1 (right).

Here, *K* is the dissociation constant for the TF and the promoter sequence, and *γ* is the ratio of the degradation rate to synthesis rate for the regulated gene. Eq (1) describes two characteristic relations between [*TF*]* and [*RG*]* depending on the *K* value (Fig 3). [*RG*]* shows a strong correlation with [*TF*]* when *K* ≫ 1, corresponding to the correlation type (Fig 1b). The expression level of the regulated gene changes depending on the TF expression level; in other words, the expression level of the regulated gene is finely regulated by the TF. Conversely, when *K* ≪ 1, [*RG*]* remains at a constant level regardless of [*TF*]*, corresponding to the horizontal-distribution type (Fig 1[c]). The regulated gene is always synthesized in large excess because the binding–dissociation equilibrium is strongly shifted toward the binding state, i.e., synthesis. Therefore, fine regulation of the gene expression level is impossible, and the on/off state of the process can only be regulated when *K* ≪ 1.

In Eq (1), [*TF*]* represents the protein concentration of a TF, but in our study we have used mRNA expression data. Although protein concentration and mRNA expression data represent different biological processes, studies have shown that there is a tolerably good correlation between the two [10, 12–14]. By assuming this correlation, we can conclude that the correlation and horizontal-distribution type relations result from differences in the transcriptional regulation mechanisms. Even without this assumption, we can say that the correlation between a TF and its regulated gene indicates fine regulation, whereas the horizontal distribution indicates the absence of regulation by the TF.

Now, we define the classification criteria of the two types as follows. The scatter diagram of the correlation type is approximated by a straight line with a finite slope (0.1 < |*s*|<10) with a certain level of accuracy (*R*^2^ > 0.3). The expression levels of both a TF and its regulated gene need to change significantly (*V*_*TF*_ > 0.25 and *V*_*RG*_ > 0.25) to show such a linear correlation. On the other hand, the diagram of the horizontal-distribution type is approximated by a horizontal line (|*s*|<0.1). The TF expression level needs to change significantly compared with the expression level of its regulated gene (*V*_*TF*_ > 0.25 and *V*_*TF*_/*V*_*RG*_ > 3) and also show uniform distribution (*U*_*TF*_ ⩾ 7).

#### The vertical-distribution type

We have not elucidated the mechanism behind the vertical-distribution type (Fig 1d). It is conceivable that genes in this type are regulated not only by TFs, but also by other factors, such as translational mechanisms.

The diagram of this type is characterized by a vertical line (|*s*|>10). In contrast to the horizontal-distribution type, the expression level of the regulated gene changes significantly compared with the TF (*V*_*RG*_ > 0.25 and *V*_*TF*_/*V*_*RG*_ < 1/3) and is distributed uniformly (*U*_*RG*_ ⩾ 7). The numerical classification criteria are summarized in Table 1 (see Methods for details).

**Table 1:** Classification criteria used to classify the scatter diagrams into the four types.

	Correlation type	Horizontal-distribution type	Vertical-distribution type
Absolute value of slope (|*s*|)	0.1–10	< 0.1	> 10
Coefficient of determination (*R*^2^)	> 0.3	-	-
Variance for TF (*V*_*TF*_)	> 0.25	> 0.25	> 0
Variance for regulated gene (*V*_*RG*_)	> 0.25	> 0	> 0.25
*V*_*TF*_ /*V*_*RG*_	-	> 3	< 1/3
Uniformity count for TF (*U*_*TF*_)	-	⩾ 7	-
Uniformity count for regulated gene (*U*_*RG*_)	-	-	⩾ 7

### Assignment of the regulated genes to the four types using the classification criteria

After classifying the nearly 280,000 diagrams into the four types (Fig 1), we assigned one correlation type to each regulated gene. It should be noted that one TF can regulate multiple genes and one gene can be regulated by multiple TFs. In addition, one regulated gene can be classified into different types depending on the experimental conditions or cellular states. To avoid ambiguous classifications, we defined a logical rule (see Methods) and assigned one type for each regulated gene depending on the GDS.

**Fig 4:**
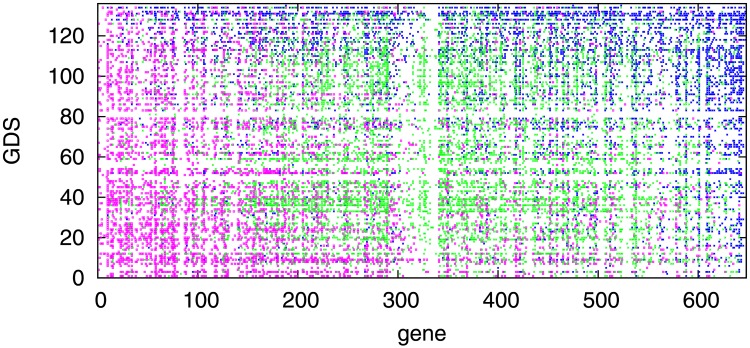
The classification results for all 647 regulated genes and for all 135 GEO DataSets. Blue represents the correlation type, magenta represents the horizontal-distribution type, green represents the vertical-distribution type, and the white areas with no points represent the no-change type or unclassified. Elements on both axes were arranged in the order of the descending proportion of the horizontal-distribution type.

Each regulated gene was classified into different relation types depending on the GDS, as shown in Fig 4. However, some genes were classified into one definite type in most GDS. To fix the type for each gene, we integrated the results from the 135 GDS by selecting a majority type from among the correlation, horizontal-distribution, and vertical-distribution types. We ignored the no-change type because our aim was to study how gene expression levels are controlled through the TF–regulated gene correlation. The no-change type occurs when there is no need to change expression levels under the experimental condition of a GDS. For example, *PSMB9* was assigned into the correlation type as 53 GDS showed the correlation and only 3 GDS showed the vertical-distribution type (Table 2). In a similar way, *CTNNB1* was assigned into the horizontal-distribution type and *CCK* was assigned into the vertical-distribution type according to the selecting a majority type rule (Table 2). Using the classification criteria, we successfully classified most of the 647 regulated genes: 111 into the correlation type, 178 into the horizontal-distribution type, and 318 into the vertical-distribution type; 40 genes could not be classified because they fell into two majority types (S2 Table).

**Table 2:** The assigned type and the numbers of GDS classified into each type are shown for regulated genes used in Fig 1.

		number of GDS showed gene			
gene	assigned type	no-change type	correlation type	horizontal-distribution type	vertical-distribution type
*PSMB9*	Correlation	79	53	0	3
*CTNNB1*	Horizontal-distribution	117	4	13	1
*CCK*	Vertical-distribution	90	3	6	36

### Gene functions of the regulated genes in three types of scatter diagrams

**Fig 5:**
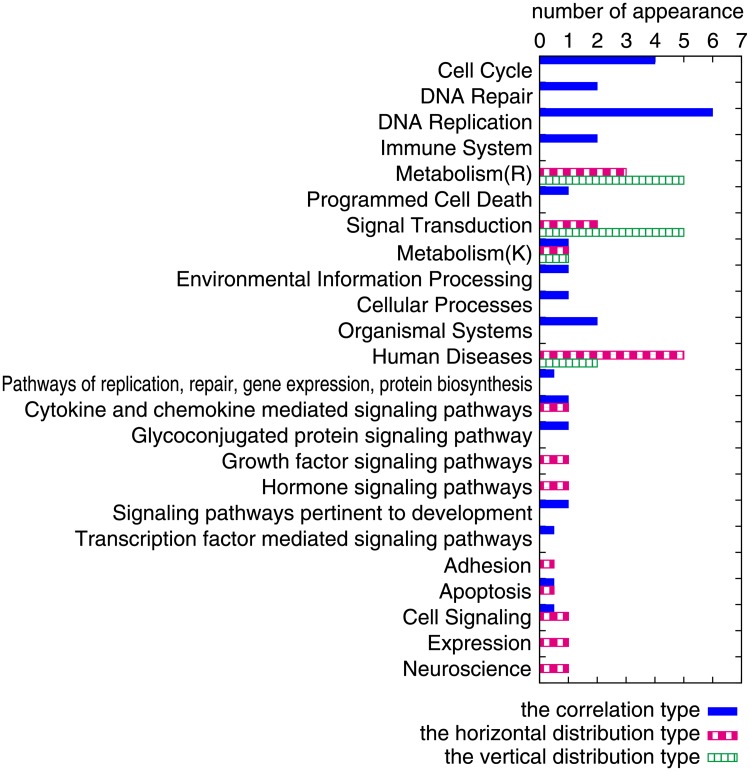
Analysis of pathways of gene functions in three types of scatter diagrams at the 1% level of significance. The number of appearances (X-axis) is shown for each hierarchical framework denoted by the top-class entity (Y-axis). Only the hierarchical frameworks that contain at least one significant pathway are shown from four pathway databases: REACTOME (from *Cell Cycle* to *Signal Transduction*), KEGG (from *Metabolism(K)* to *Human Diseases*), the Pathway Interaction Database (from *Pathways of replication, repair, gene expression, and protein biosynthesis* to *Transcription factor-mediated signaling pathways*), and the BioCarta (from *Adhesion* to *Neuroscience*). If a pathway belonged to more than one (*n*_*p*_) hierarchical framework, we counted 1/*n*_*p*_ for each.

We performed pathway analysis of the gene functions in the correlation, horizontal-distribution and vertical-distribution types using the curated gene sets in the Canonical pathways (C2:CP) from the Molecular Signatures Database (MSigDB; http://www.broadinstitute.org/gsea/msigdb) [20] with the hypergeometric test at the 1% level of significance (see Methods). We obtained 25 significant pathways for the correlation type, 19 significant pathways for the horizontal-distribution type, and 14 significant pathways for the vertical-distribution type (S3 Table). To compare the different relation types, we categorized the pathways according to the hierarchical framework by denoting a pathway by the top-class entity of its hierarchical framework (Fig 5).

To our surprise, we found that some of the genes in each relation type were associated with type-specific functions: cellular regulation (e.g., *Cell Cycle* and *DNA Replication*) for the correlation type, *Human Diseases* for the horizontal-distribution type, and *Metabolism* or *Signal Transduction* for the vertical-distribution type. It is interesting that serious diseases, such as cancers, Parkinson’s disease, Alzheimer’s disease, and Huntington’s disease, were observed in the horizontal-distribution type, i.e., the degradation dominant type. The implications of this observation are considered in the *Discussion*. To summarize, the scatter diagrams for the TF–regulated gene pairs were characterized systematically according to the functions of the regulated genes. The results indicate that the mechanisms that regulate gene expression levels correspond to gene functions at the whole-cell level.

## Discussion

In this work, we studied regulatory patterns of gene expression where TFs regulate the transcription process in a fine or on/off manner. We drew scatter diagrams for TF–regulated gene pairs using publicly available DNA microarray data and classified the diagrams into four types based on our simple mathematical model of a transcription process. We also performed pathway analysis and found that the relation types could be linked to the gene functions. Genes related to cellular regulation processes belonged to the correlation type, which indicates fine regulation of the transcription rate. Genes related to diseases belonged to the horizontal-distribution type, which indicates on/off regulation of the transcription process. Genes related to metabolism or signal transduction belonged to the vertical-distribution type, where the regulatory mechanism is unclear. These findings imply that the regulatory mechanisms for transcription processes are determined not arbitrarily but systematically depending on gene function, and pointing to the presence of a whole-cell regulatory mechanism.

Here, we classified 647 regulated genes into four classification types. To our surprise, the correlation type (fine regulation of a transcription process) was observed less frequently than we expected (less than 20% of the genes), although such fine regulation has often been assumed. The regulatory mechanism of the correlation type requires the expression levels of both the TF and the regulated gene to be fine-tuned to specific values depending on cellular states. Such fine-tuning would be a challenging task for many genes and would be impossible on a whole-cell level. This might explain why the correlation type was rarer than expected.

We used mRNA expression data in this study because of technical limitations. The final product of gene expression processes is usually a protein, and we plan to study protein data in the near future. Studies on the human proteome are still a developing field [11, 21, 22], and there are several challenges for protein quantification and for the organization of such data into databases [10, 12]. The mechanisms regulating protein abundance are more complicated than for mRNA, but in the simplest terms, protein abundance can be regulated via the balance between synthesis rates and degradation rates. The analytical method that we developed here could also be applied to protein data. Analyses of protein data will shed more light on the mechanisms that govern the transfer of the quantitative property of genomic information.

Finally, it is worth discussing genes classified in the horizontal-distribution type (on/off regulation of a transcription process). They are often over-expressed; therefore, it can be hypothesized that the abundance of the encoded protein needs to be controlled by degradation to the appropriate levels after excessive synthesis. Regulation through degradation is not as common as the regulation via synthesis [23, 24]. Examples of regulation through degradation include the well-studied proteins p53, which is a tumor suppressor that also regulated the cell cycle [25–27], and *β*-catenin, which is a signal transducer in the Wnt signaling pathway that also regulates cell-cell adhesion [28]. In addition, some reports have indicated that HIF-1*α*, a TF that responds to a shortage of oxygen [29], may be regulated through degradation. In the present study, p53 and *β*-catenin were classified into the horizontal-distribution type during our analysis, but we did not have sufficient data to classify HIF-1*α*.

It should also be noted, that the pathway analysis showed that genes in the horizontal-distribution type were associated with diseases, especially serious diseases such as cancer, Parkinson’s disease, and Alzheimer’s disease, and both p53 and *β*-catenin have been strongly implicated in cancer [30]. These diseases are caused by the abnormal accumulation of some proteins [31], and for good health, their abundance needs to be kept at low levels. Interestingly, we found that their abundance was regulated not through synthesis but through degradation after over-expression, although such regulation seems irrational and risky in cases when protein accumulation causes diseases. We expect that our future theoretical research will give some clues to such inconsistencies.

## Methods

### Preparation of DNA microarray data sets

We used the DNA microarray data from GDS as the expression data in this study. First, we normalized the expression data and removed measurement specificity, generally involving different DNA microarray instruments, to compare the different GDS. Several normalization procedures are available and each has its own advantages [32–37]. In this study, we needed a general-purpose method applicable to various measurement platforms and used a popular method, Z scores [33, 37, 38], as follows.

For each sample in a GDS, we first transformed the original expression data given as {*x*_1_,*x*_2_,…,*x*_*s*_} to the *log*-scale,

$$\begin{array}{c} \{\text{ln}\left(x_{1}\right),\text{ln}\left(x_{2}\right),\cdots ,\text{ln}\left(x_{s}\right)\}. \end{array}$$ (2)

Then, we normalized the values using the Z-score method by defining

$$\begin{array}{c} E=\frac{1}{s}\sum_{i=1}^{s}\text{ln}\left(x_{i}\right),V=\sqrt{\frac{s}{s-1}\left\{\frac{1}{s}\sum_{i=1}^{s}{\left(\text{ln}\left(x_{i}\right)\right)}^{2}-E^{2}\right\}}. \end{array}$$ (3)

The normalized value is given as

$$\begin{array}{c} \frac{\text{ln}(x_{i})-E}{V} \end{array}$$ (4)

for every *x*_*i*_.

### TF-regulated gene scatter diagrams

We constructed scatter diagrams for expression levels of each TF (*TF*_*i*_) and its regulated gene (*RG*_*i*_) from a GDS. Suppose a GDS contains *N*_*i*_(⩾50) samples, then the diagram has *N*_*i*_ points as explained below. When a sample had only one data point for *TF*_*i*_ (*RG*_*i*_), we used this value for plotting, and when a sample contained more than one data point for *TF*_*i*_ (*RG*_*i*_), we used the average value. Thus, one sample yielded one point, and the diagram had *N*_*i*_ points in total.

One GDS normally contains subclasses such as an experimental group and a control group. It could be that each subclass produces a different domain structure in the diagram and falsely represents an imaginary correlation. Namely, if the samples in one subclass show smaller *TF*_*i*_ and *RG*_*i*_ and the samples in another subclass show higher *TF*_*i*_ and *RG*_*i*_ because of the experimental conditions, a correlation may be observed between *TF*_*i*_ and *RG*_*i*_ even if there is no real correlation. We confirmed that such imaginary correlations appeared rarely and did not influence the results.

### TF-regulated gene binding transcription model

We considered a general and simple mathematical model of a transcription process. We analyzed two situations: TF promoting gene expression (up-regulation), and TF suppressing gene expression (down-regulation). First, we explain the up-regulation case in detail and next the down-regulation case in brief.

For the up-regulation case, suppose a TF molecule stochastically binds to or dissociates from a promoter sequence, and transcription takes place only when the TF binds to the sequence. Then, *P*_*b*_ is the probability of the TF’s binding to the promoter sequence, and is defined as a fraction of bound TF molecules among all TF molecules. By assuming that the binding process and dissociation process are in equilibrium, we get the following equation:

$$\begin{array}{c} k_{b}\left[TF\right]\left(1-P_{b}\right)=k_{u}P_{b}. \end{array}$$ (5)

Here, [*TF*] represents the TF concentration, and *k*_*b*_ and *k*_*u*_ are the reaction coefficients for the binding and dissociation processes, respectively. The left side of Eq (5) represents the reaction rate of the TF binding process proportional to the product of the TF concentration ([*TF*]) and the unbound promoter sequence (1−*P*_*b*_). On the other hand, the right side represents the TF dissociation reaction rate regulated by the bound promoter sequence(*P*_*b*_). From Eq (5), we obtain

$$\begin{array}{c} P_{b}=\frac{\left[TF\right]/K}{1+\left[TF\right]/K} \end{array}$$ (6)

where the dissociation constant *K* = *k*_*u*_/*k*_*b*_. Because transcription occurs only when the TF binds to the promoter region, the mRNA synthesis rate of the regulated gene should be proportional to *P*_*b*_. Therefore, we can write the time dependence of the expression of the regulated gene mRNA ([*RG*]) as

$$\begin{array}{c} \frac{d\left[RG\right]}{dt}=a\frac{\left[TF\right]/K}{1+\left[TF\right]/K}-b\left[RG\right]. \end{array}$$ (7)

Here, *a* and *b* are reaction coefficients for the synthesis and degradation. By considering a steady state of Eq (7), $\frac{d\left[RG\right]*}{dt}=0$, we finally obtain the steady-state mRNA level of the regulated gene ([*RG*]*) as a function of the steady-state TF concentration ([*TF*]*) as shown in Eq (1),

$$\begin{array}{c} {\left[RG\right]}^{*}=\frac{1}{\gamma }\frac{{\left[TF\right]}^{*}/K}{1+{\left[TF\right]}^{*}/K}. \end{array}$$ (1)

For the down-regulation case, we obtain the following equation from a similar analysis except that the production rate is proportional to the dissociation probability (1−*P*_*b*_):

$$\begin{array}{c} \frac{d\left[RG\right]}{dt}=a^{\prime }\left(1-\frac{\left[TF\right]/K^{\prime }}{1+\left[TF\right]/K^{\prime }}\right)-b^{\prime }\left[RG\right] \end{array}$$ (8)

and therefore

$$\begin{array}{c} {\left[RG\right]}^{*}=\frac{a^{\prime }}{b^{\prime }}\frac{1}{1+{\left[TF\right]}^{*}/K^{\prime }}. \end{array}$$ (9)

Eq (9) describes the same two types of characteristic behaviors as Eq (1) depending on the dissociation constant *K*′, although [*RG*]* shows a strong negative correlation with [*TF*]* when *K*′ ≪ 1 and [*RG*]* remains at a constant level regardless of [*TF*]* when *K*′ ≫ 1. In the up-regulation or down-regulation cases, the correlation between a TF and the regulated gene indicates fine-tuned rate regulation, whereas the horizontal distribution indicates the absence of regulation.

### Classification criteria

We defined the criteria for classifying the scatter diagrams into the four types as follows. First, we excluded the data with *V*_*TF*_ = 0 or *V*_*RG*_ = 0 because they probably originate from a measurement flaw. It is virtually impossible for all the samples in a GDS to show exactly the same expression level of a gene. We also assumed that the expression level of a TF (or a regulated gene) changed significantly when *V*_*TF*_ > 0.25 (*V*_*RG*_ > 0.25), whereas it is constant, albeit with small fluctuations, when *V*_*TF*_ ⩽ 0.25 (*V*_*RG*_ ⩽ 0.25). After that, we classified those with 0.1 < |*s*|<10 and *R*^2^ > 0.3 into the correlation type when *V*_*TF*_ > 0.25 and *V*_*RG*_ > 0.25. We then classified diagrams with |*s*|<0.1, *V*_*TF*_ > 0.25 (*V*_*RG*_ > 0), *V*_*TF*_/*V*_*RG*_ > 3, and *U*_*TF*_ ⩾ 7 into the horizontal-distribution type, and diagrams with |*s*|>10, *V*_*RG*_ > 0.25 (*V*_*TF*_ > 0), *V*_*TF*_/*V*_*RG*_ < 1/3, and *U*_*RG*_ ⩾ 7 into the vertical-distribution type. The remaining diagrams were assigned to the no-change type. The baseline values used here were set arbitrarily, but the discussion will not change if the values are changed to some extent.

### Logical rule for combining multiple TFs

In many cases, TFs and regulated genes do not have a one-to-one correspondence. When a regulated gene has several TFs, some of the TFs finely regulate the transcription process whereas others simply switch the process on or off. The former TF–regulated gene pairs may match the correlation type, whereas the latter often correspond to the vertical-distribution type. In such a mixed case, the regulated gene should be classified into the correlation type not into the vertical-distribution type. Similarly, when TF–regulated gene pairs match the horizontal-distribution type and others correspond to the no-change type, the regulated gene should be classified into the horizontal-distribution type. Using these rules, we classified every regulated gene as follows.

Suppose a gene has *M* TFs (*M* ⩾ 1) and among them, *M*_*n*_ TFs are of the no-change type, *M*_*c*_ of the correlation type, *M*_*h*_ of the horizontal-distribution type, and *M*_*v*_ TFs are of the vertical-distribution type. When *M*_*h*_ > *M*_*c*_ and *M*_*v*_, the regulated gene is classified into the horizontal-distribution type; when *M*_*v*_ ⩾ *M*_*h*_ ⩾ *M*_*c*_ = 0, the regulated gene is classified into the vertical-distribution type; and when *M*_*c*_ > *M*_*h*_ ⩾ 0, the regulated gene is classified into the correlation type, regardless of *M*_*v*_. This is because a TF, even if it serves as a single TF, can determine the correlation type as explained above. We classified a regulated gene into the no-change type only when *M*_*n*_ = *M*. For the remaining cases, we aborted the classification because there was not sufficient evidence. We thus assigned one classification type to each regulated gene depending on the GDS.

### The hypergeometric test

We determined whether a list of genes (genes of each relation type) over-represents a biological process (gene sets for a pathway from MsigDB) using the hypergeometric test. Suppose we listed *n* genes from a total of *N* genes; i.e., we selected *n* genes without replacement from the *N* genes. *M* genes among the total of *N* genes are involved in the biological process, and *m* genes among the listed *n* genes are involved in the same process. Then, the probability distribution of *m* (*p*(*m*)) is described by the hypergeometric distribution as

$$\begin{array}{c} p\left(m\right)=\frac{\left(\frac{M}{m}\right)\left(\frac{N-M}{n-m}\right)}{\left(\frac{N}{n}\right)}. \end{array}$$ (10)

Our goal is to determine whether the case of *m* genes being involved in the biological process (out of the *n* listed genes) is statistically significant or happened by chance. Because we are testing whether our gene set corresponds to over-representation, the hypergeometric *p* value (*p*) is calculated as the probability of random involvement of *m* or more genes in the biological process (out of *n* genes) and is expressed as

$$\begin{array}{c} p=\sum_{x=m}^{n}p\left(x\right)=\sum_{x=m}^{n}\frac{\left(\frac{M}{x}\right)\left(\frac{N-M}{n-x}\right)}{\left(\frac{N}{n}\right)}. \end{array}$$ (11)

When the *p* value is less than the value we set as the level of significance (1%), we conclude that our set of genes is over-represented, i.e., the *m* genes occurred non-randomly. However, when the *p* value is greater than the threshold value, we conclude that the *m* genes are selected by chance.

## Supporting information

S1 Table List of the GEO DataSets used in this study. Title and the number of samples are shown for each DataSet from the Gene Expression Omnibus (GEO) at the NCBI.
(PDF)

S2 Table Assignment of genes into the four types. For each assigned type, gene names and the numbers of GDS classified into each type are shown. The no data column shows the number of GDS data was not available.
(PDF)

S3 Table List of the selected pathways from pathway analysis. The category (hierarchical framework), the pathway name, and the name of involved genes are shown for each selected pathway.
(PDF)

S1 Fig Workflow of whole study. (EPS)
