# Supplementary material for: Relationship between regulatory pattern of gene expression level and gene function
Source: PLoS One. 2017 May 11;12(5):e0177430. doi: 10.1371/journal.pone.0177430 (PMC5426767; doi:10.1371/journal.pone.0177430)
Supplement: S1 Table — Title and the number of samples are shown for each DataSet from the Gene Expression Omnibus (GEO) at the NCBI. (PDF) [file pone.0177430.s001.pdf]

S1 Table : List of the GEO DataSets used in this study

DataSet	Title	number of samples
GDS181	Large-scale analysis of the human transcriptome (HG-U95A)	84
GDS531	Multiple myeloma and bone lesions	173
GDS534	Smoking-induced changes in airway transcriptome	75
GDS596	Large-scale analysis of the human transcriptome (HG-U133A)	158
GDS711	Juvenile rheumatoid arthritis expression profiles in mononuclear cells	57
GDS724	Kidney transplant rejection expression profiling	62
GDS830	Alternative pre-mRNA splicing in various tissues and cell lines (Rosetta/Merck Splicing Chip 2)	50
GDS968	Radiation therapy toxicity association with abnormal transcriptional response to DNA damage	171
GDS1048	Lymphoblastoid cell lines from CEPH/Utah families	167
GDS1059	Acute myeloid leukemia response to chemotherapy	58
GDS1067	Plasma cell dyscrasias	52
GDS1209	Sarcoma and hypoxia	54
GDS1220	Malignant pleural mesothelioma	54
GDS1284	Multiple myeloma molecular classification	50
GDS1375	Cutaneous malignant melanoma	70
GDS1402	Various normal pure cell cultures	61
GDS1412	Hormone replacement therapy effect on whole blood	89
GDS1449	HIV-1 infection effect on peripheral blood mononuclear cells	87
GDS1479	Carcinoma in situ lesions of the urinary bladder	60
GDS1597	Coronary artery atherosclerosis	51
GDS1615	Ulcerative colitis and Crohn's disease comparison: peripheral blood mononuclear cells	127
GDS1627	Breast cancer cell lines response to chemotherapeutic drugs: time course	83
GDS1813	Glial brain tumors	53
GDS1815	High-grade gliomas (HG-U133A)	100
GDS1816	High-grade gliomas (HG-U133B)	100
GDS1956	Various muscle diseases (HG-U133A)	121
GDS1962	Glioma-derived stem cell factor effect on angiogenesis in the brain	180
GDS1975	Gliomas of grades III and IV (HG-U133A)	85
GDS1976	Gliomas of grades III and IV (HG-U133B)	85
GDS2106	Lymphoblastoid cell lines from various CEPH pedigrees	100
GDS2113	Pheochromocytomas of various genetic origins	75
GDS2118	Myelodysplastic syndromes: CD34+ cells	66
GDS2190	Bipolar disorder: dorsolateral prefrontal cortex	61
GDS2255	Transmigrated neutrophils in the alveolar space of endotoxin-exposed lung	58
GDS2362	Presymptomatic and symptomatic malaria: peripheral blood mononuclear cells	71
GDS2373	Squamous cell lung carcinomas	130
GDS2519	Early-stage Parkinson's disease: whole blood	105
GDS2545	Metastatic prostate cancer (HG-U95A)	171
GDS2546	Metastatic prostate cancer (HG-U95B)	167
GDS2547	Metastatic prostate cancer (HG-U95C)	164
GDS2626	Epidermal growth factor and heregulin effect on breast cancer cell line: dose response and time course	57
GDS2643	Waldenstrom's macroglobulinemia: B lymphocytes and plasma cells	56
GDS2733	Cytosine arabinoside effect on Ewing's sarcoma cell line: time course and dose response	68
GDS2736	Malignant fibrous histiocytoma and various soft tissue sarcomas	105
GDS2767	Blood response to various beverages: time course	108
GDS2771	Large airway epithelial cells from cigarette smokers with suspect lung cancer	192
GDS2819	Leukemic white blood cells and various RNA preparation protocols	99
GDS2855	Various muscle diseases (HG-U133B)	119
GDS2926	Megakaryocytic differentiation: time course	77
GDS2947	Colorectal adenoma formation	64
GDS2954	Mycobacterium tuberculosis-derived lipopeptide effect on monocytes and dendritic cells: time course	50
GDS2960	Marfan syndrome: cultured skin fibroblasts	101
GDS3017	Cervical cancer response to chemoradiotherapy	156
GDS3057	Acute myeloid leukemia	64
GDS3113	Various normal tissues	96
GDS3116	Letrozole effect on breast cancer tumors	116
GDS3233	Cervical cancer tumorigenesis	61
GDS3257	Cigarette smoking effect on lung adenocarcinoma	107
GDS3268	Colon epithelial biopsies of ulcerative colitis patients	202
GDS3289	Prostate cancer progression at the cellular level	104
GDS3308	Cytogenetically normal acute myeloid leukemia_training set (HG-U133B)	163
GDS3312	Cytogenetically normal acute myeloid leukemia_training set (HG-U133A)	163
GDS3324	Stromal cells and invasive breast cancer development	66
GDS3325	Air pollution exposure effect on children and adults: peripheral blood	71
GDS3326	Periodontal therapy effect on peripheral blood monocytes: time course	59
GDS3329	Cytogenetically normal acute myeloid leukemia_test set	79
GDS3345	Various mental disorders: postmortem brains	50

DataSet	Title	number of samples
GDS3356	Bronchopulmonary dysplasia: premature newborn umbilical cords	54
GDS3416	Relaxation response practice effect on blood	72
GDS3432	Osteosarcoma cell line response to activation of specific glucocorticoid receptor alpha isoforms: time course	60
GDS3459	Frontotemporal lobar degeneration with ubiquitinated inclusions and progranulin mutations: various brain regions	56
GDS3494	Cigarette smoke of light flavor brand effect on bronchial epithelial cells in vitro: time course	52
GDS3499	TREM-1 activation effect on monocytes in vitro	62
GDS3516	Nodular lymphocyte-predominant Hodgkin lymphoma: lymphocytic and histiocytic cells	67
GDS3517	Oncogenic NRAS depletion effect on melanoma cell lines: time course	51
GDS3539	Psoriasis	82
GDS3553	Monocytes and macrophages (RNG/MRC)	96
GDS3603	Renal cancer response to rapamycin analog CCI-779 treatment: time course	79
GDS3627	Non-small lung cancer subtypes: adenocarcinoma and squamous cell carcinoma	58
GDS3640	Copper effect on liver cell line: dose response and time course	98
GDS3646	Celiac disease: primary leukocytes	132
GDS3690	Atherosclerotic Coronary Artery Disease: circulating mononuclear cell types	153
GDS3709	Cigarette smoke effect on the oral mucosa	79
GDS3713	Smoking effect on B lymphocytes in females	79
GDS3715	Insulin effect on skeletal muscle	110
GDS3829	Fludarabine, cyclophosphamide, and rituximab chemoimmunotherapy effect on chronic lymphocytic leukemia	50
GDS3837	Non-small cell lung carcinoma in female nonsmokers	120
GDS3842	Transcription factor-induced pluripotent stem cells	51
GDS3874	Diabetic children: peripheral blood mononuclear cells (U133A)	117
GDS3875	Diabetic children: peripheral blood mononuclear cells (U133B)	117
GDS3884	Type 2 diabetic and insulin-resistant but normoglycemic cohorts: skeletal muscle	50
GDS3885	Glioblastoma stem-like cell lines, glioblastomas, and glioma cell lines	92
GDS3898	Social isolation effect on peripheral blood mononuclear cells	93
GDS3916	Recombinant human growth hormone effect on lactating mammary gland: time course	139
GDS3919	Severe influenza A infection: whole blood	81
GDS3929	Tobacco smoke effect on maternal and fetal cells	183
GDS3952	Breast cancer patients: peripheral blood mononuclear cells	162
GDS3966	Melanoma metastasis	83
GDS4056	ER-positive/HER2-negative and ER-negative/HER2-negative breast cancer biopsies (USO-02103 cohort)	61
GDS4057	ER-positive/HER2-negative and ER-negative/HER2-negative breast cancer biopsies (MDACC/IGR cohort)	103
GDS4088	Variable breast cancer tumor sample collection methods	86
GDS4102	Pancreatic Tumor and Normal tissue samples	52
GDS4103	ICF cohort: Whole-tissue pancreatic ductal adenocarcinoma	78
GDS4109	Recurrent and non-recurrent prostate cancer primary tumors	79
GDS4129	Endoplasmic reticulum stress effect on lymphoblastoid cells of unrelated individuals	120
GDS4130	Endoplasmic reticulum stress effect on lymphoblastoid cells of monozygotic twins	104
GDS4145	Subcutaneous Interferon-beta-1b treatment in relapsing-remitting multiple sclerosis (U133 A): peripheral mononuclear blood cells	125
GDS4146	Subcutaneous Interferon-beta-1b treatment in relapsing-remitting multiple sclerosis (U133 B): peripheral mononuclear blood cells	125
GDS4167	Chronic lymphocytic leukemia: peripheral blood B cells (HG-U133A)	52
GDS4168	Chronic lymphocytic leukemia: peripheral blood B cells (HG-U133B)	52
GDS4176	Chronic lymphocytic leukemia: peripheral blood, bone marrow and lymph node matched samples	62
GDS4222	Classic Hodgkins lymphoma diagnostic lymph-node biopsies	130
GDS4228	HIV infection and Antiretroviral Therapy effects on mitochondria in various tissues	166
GDS4238	Bronchial epithelial cell response to influenza virus, viral RNA and interferon-beta: time course	169
GDS4262	Oxidized low-density lipoprotein effect on LOX-1 overexpressing aortic endothelial cell line HAECT: time course	54
GDS4265	Chronic obstructive pulmonary disease in ex-smokers: sputum	143
GDS4266	Tolerant renal transplant recipients: peripheral whole blood	58
GDS4278	Acute myeloid leukemia with CEBPA mutations [AMLSG cohort]: mononuclear cells	154
GDS4282	Clear-cell renal cell carcinoma tumors and tumorgrafts deficient for tumor suppressor BAP1 or PBRM1	76
GDS4284	Chronic lymphocytic leukemia response to antisense Bcl-2 inhibitor SPC2996: peripheral blood	56
GDS4296	NCI-60 cancer cell line panel	174
GDS4299	Early T-cell precursor acute lymphoblastic leukemia	52
GDS4318	Single nucleotide polymorphism rs1333049 from the 9p21.3 coronary artery disease risk locus: normal heart	108
GDS4336	Pancreatic ductal adenocarcinoma tumor and adjacent non-tumor tissue	90
GDS4337	Type 2 diabetic and hyperglycemic pancreatic islets	63
GDS4358	Two types of HIV-associated neurocognitive impairment: brain regions	72
GDS4379	Colorectal cancer tumors	62
GDS4381	Patient-derived colorectal cancer explants	64
GDS4393	Unresectable colorectal cancer primary or metastatic lesions: training set	54
GDS4395	External beam radiation therapy effect on prostate cancer patients: peripheral white blood cells	80
GDS4404	Facioscapulohumeral muscular dystrophy: bicep and deltoid muscles	50
GDS4412	Dietary fatty acid composition effect on vastus lateralis muscle	56
GDS4431	Autistic children and their father's age: peripheral blood lymphocytes	146
GDS4471	Medulloblastomas in children	76
GDS4513	Clinical outcome of stage UICC II colon cancer patients	53
