# Supplementary material for: Relationship between regulatory pattern of gene expression level and gene function
Source: PLoS One. 2017 May 11;12(5):e0177430. doi: 10.1371/journal.pone.0177430 (PMC5426767; doi:10.1371/journal.pone.0177430)
Supplement: S2 Table — For each assigned type, gene names and the numbers of GDS classified into each type are shown. The no data column shows the number of GDS data was not available. (PDF) [file pone.0177430.s002.pdf]

S2. Table : Assignment of genes into the four types

The correlation-type (111genes)

assigned_type	gene_name	number of GDS				
		no-change type	correlation type	horizontal-distribution type	vertical-distribution type	no data
correlation	ATF3	63	64	2	5	1
correlation	PSMB9	79	53	0	3	0
correlation	STAT1	83	43	4	5	0
correlation	CCL5	56	48	7	23	1
correlation	WARS	97	33	1	4	0
correlation	CD14	57	45	4	28	1
correlation	CXCL8	37	52	4	42	0
correlation	IDO1	85	34	2	14	0
correlation	CD8A	87	33	2	13	0
correlation	ITGB2	98	29	1	6	1
correlation	IFITM1	110	25	0	0	0
correlation	TYMP	102	27	0	6	0
correlation	PSMB10	108	25	1	1	0
correlation	PFKFB3	91	30	3	11	0
correlation	DDIT3	95	28	7	5	0
correlation	AURKB	103	25	0	7	0
correlation	HLA-E	96	26	13	0	0
correlation	ICAM1	59	38	27	10	1
correlation	PTPN6	88	28	12	7	0
correlation	CD3E	96	24	4	10	1
correlation	VIM	70	33	19	13	0
correlation	TNFRSF10B	105	21	4	4	1
correlation	HLA-G	84	27	17	6	1
correlation	CD19	113	17	1	4	0
correlation	CLDN7	116	16	1	2	0
correlation	PMAIP1	110	18	3	4	0
correlation	CTSL	85	26	8	16	0
correlation	MYD88	109	18	4	4	0
correlation	FAS	71	30	23	10	1
correlation	LY96	111	17	2	5	0
correlation	CDKN1B	84	25	21	3	2
correlation	CDC25A	103	19	8	5	0
correlation	CD274	112	16	0	7	0
correlation	LCK	101	19	4	11	0
correlation	MYH9	120	12	3	0	0
correlation	PLAUR	84	24	9	18	0
correlation	CCL4	96	20	1	18	0
correlation	CD86	113	14	4	4	0
correlation	GCLM	106	16	4	9	0
correlation	ITGAM	103	17	10	5	0
correlation	SLC11A1	93	20	17	4	1
correlation	ACTB	105	16	12	2	0
correlation	ODC1	105	16	6	8	0
correlation	SPTA1	114	13	6	2	0
correlation	CXCL12	116	12	0	7	0
correlation	BAX	97	18	8	12	0
correlation	CASP8	117	11	4	3	0
correlation	POLD1	120	10	1	4	0
correlation	CAD	125	8	2	0	0
correlation	CDH13	119	10	1	5	0
correlation	CDKN1A	52	32	19	31	1
correlation	C3AR1	100	16	10	9	0
correlation	BID	120	9	5	1	0

correlation	BRCA1	83	21	17	13	1
correlation	IGF1	123	8	0	4	0
correlation	SERPINE1	48	33	24	30	0
correlation	PIK3CA	126	7	1	1	0
correlation	PLAU	59	28	17	27	4
correlation	TFAM	123	8	4	0	0
correlation	TGFB1	108	13	9	5	0
correlation	IGFBP5	113	11	6	5	0
correlation	NFKB2	128	6	0	1	0
correlation	YWHAE	122	8	4	1	0
correlation	PTTG1	98	16	9	12	0
correlation	GCLC	115	10	6	4	0
correlation	NCAM1	130	5	0	0	0
correlation	RHO	127	6	2	0	0
correlation	TGFBR1	124	7	0	4	0
correlation	NR1I2	124	7	0	4	0
correlation	ANGPT2	111	11	4	9	0
correlation	FTH1	126	6	2	1	0
correlation	ITGB7	126	6	3	0	0
correlation	PCNA	111	11	4	9	0
correlation	TBXAS1	123	7	3	2	0
correlation	ITGAV	98	15	8	14	0
correlation	LAT	131	4	0	0	0
correlation	GPR132	116	9	6	4	0
correlation	CCNE1	114	9	6	5	1
correlation	CHEK1	127	5	0	3	0
correlation	DDB2	130	4	1	0	0
correlation	F7	112	10	8	5	0
correlation	FDFT1	121	7	5	2	0
correlation	FPGS	127	5	1	2	0
correlation	SPN	118	8	5	4	0
correlation	PAICS	118	8	3	6	0
correlation	BSG	117	8	3	7	0
correlation	GPD2	126	5	2	2	0
correlation	PDGFB	111	10	9	5	0
correlation	TYMS	120	7	3	5	0
correlation	VWF	111	10	5	9	0
correlation	SLC28A1	132	3	0	0	0
correlation	ACADM	113	9	7	6	0
correlation	ACAT1	125	5	3	2	0
correlation	FGFR4	107	11	8	9	0
correlation	MC1R	125	5	4	1	0
correlation	RAD51	131	3	0	1	0
correlation	RARA	125	5	4	1	0
correlation	TLN1	131	3	1	0	0
correlation	AKT1	130	3	0	2	0
correlation	ABCC1	124	5	3	3	0
correlation	POLA1	127	4	1	3	0
correlation	PSEN2	124	5	4	2	0
correlation	RRAS	127	4	1	3	0
correlation	BBC3	127	4	3	1	0
correlation	ADORA2A	132	2	1	0	0
correlation	CALCA	129	3	2	1	0
correlation	TFRC	114	8	6	7	0
correlation	WAS	126	4	3	2	0
correlation	CGB	131	2	1	1	0
correlation	CCR3	125	4	3	3	0
correlation	TICAM1	131	2	1	1	0

The horizontal-distribution type (178 genes)

assigned_type	gene_name	number of GDS				
		no-change type	correlation type	horizontal-distribution type	vertical-distribution type	no data
horizontal	SKP1	56	3	76	0	0
horizontal	TBXA2R	57	11	64	2	1
horizontal	HNF4A	72	7	50	3	3
horizontal	EGFR	61	12	54	7	1
horizontal	IMPDH2	81	1	46	5	2
horizontal	OPRM1	71	2	50	12	0
horizontal	BCL2	66	16	50	2	1
horizontal	ESR1	70	11	48	5	1
horizontal	GNRHR	70	2	47	15	1
horizontal	VDR	62	20	49	3	1
horizontal	SOD1	82	10	42	1	0
horizontal	CYP19A1	50	6	51	26	2
horizontal	GNAI2	64	16	45	5	5
horizontal	SLC6A2	93	4	37	1	0
horizontal	FASLG	71	7	42	15	0
horizontal	GNAQ	100	1	32	2	0
horizontal	DBH	61	7	43	22	2
horizontal	ELK1	105	2	28	0	0
horizontal	SLC6A3	98	3	30	4	0
horizontal	GAPDH	93	8	30	3	1
horizontal	PSEN1	81	16	33	4	1
horizontal	SLC12A4	103	2	25	5	0
horizontal	NAT1	74	8	33	16	4
horizontal	NFKB1	81	18	32	4	0
horizontal	CYP2C9	91	7	28	8	1
horizontal	SLC19A1	96	8	26	3	2
horizontal	WEE1	101	9	25	0	0
horizontal	FLT1	96	7	26	5	1
horizontal	MMP2	71	10	33	17	4
horizontal	SLC9A2	93	4	27	11	0
horizontal	GH1	84	11	29	9	2
horizontal	GSTP1	82	11	30	12	0
horizontal	TSHB	101	5	23	5	1
horizontal	APEX1	113	2	19	1	0
horizontal	LDLR	79	15	30	10	1
horizontal	CYP2A13	100	2	23	10	0
horizontal	ESRRA	115	2	18	0	0
horizontal	ITGB1	118	0	17	0	0
horizontal	FYN	101	5	22	6	1
horizontal	RFC1	108	3	20	4	0
horizontal	ERBB2	98	9	23	5	0
horizontal	HSP90AA1	110	5	19	1	0
horizontal	FGFR2	111	3	18	2	1
horizontal	CSNK1A1	114	4	17	0	0
horizontal	RARB	105	4	20	6	0
horizontal	COL2A1	88	6	25	15	1
horizontal	CYP2B6	101	8	21	5	0
horizontal	YBX1	101	12	21	1	0
horizontal	ACTA2	82	11	27	15	0
horizontal	CLDN18	106	0	19	10	0
horizontal	CYP3A4	87	14	25	9	0
horizontal	SRSF2	114	5	16	0	0
horizontal	SREBF1	114	3	16	2	0
horizontal	ALMS1	114	3	16	2	0

horizontal	CD8B	110	7	17	1	0
horizontal	CTNND1	119	1	14	1	0
horizontal	TALDO1	107	5	18	5	0
horizontal	FGF2	97	8	21	9	0
horizontal	FANCC	123	0	12	0	0
horizontal	TERF2	123	0	12	0	0
horizontal	CDK5R1	117	4	14	0	0
horizontal	CRH	86	3	24	22	0
horizontal	LAMA3	110	6	16	3	0
horizontal	FURIN	104	8	18	5	0
horizontal	NR3C1	78	19	26	11	1
horizontal	CTNNB1	117	4	13	1	0
horizontal	RPL10	116	2	13	3	1
horizontal	CDK4	116	6	13	0	0
horizontal	DHFR	107	5	16	7	0
horizontal	MAT2A	107	9	16	3	0
horizontal	PPP2CA	119	3	12	1	0
horizontal	SLC9A3	93	9	20	11	2
horizontal	TP53	55	20	33	26	1
horizontal	SLC5A6	113	7	14	1	0
horizontal	B2M	97	14	18	3	3
horizontal	CCND3	112	4	14	5	0
horizontal	LAMC1	121	2	11	1	0
horizontal	PPARA	124	0	10	1	0
horizontal	VAMP7	106	8	16	5	0
horizontal	APH1A	124	0	10	1	0
horizontal	NANOG	115	3	13	4	0
horizontal	CREB1	120	3	11	1	0
horizontal	GJB1	104	12	16	2	1
horizontal	IGF2	114	4	13	4	0
horizontal	MDM2	117	4	12	2	0
horizontal	RELA	123	0	10	2	0
horizontal	SCARB1	119	0	11	5	0
horizontal	PSMD10	119	4	11	1	0
horizontal	SDHA	122	2	10	1	0
horizontal	APP	91	6	20	18	0
horizontal	ATF2	112	6	13	4	0
horizontal	CYP2D6	124	2	9	0	0
horizontal	FANCE	124	1	9	1	0
horizontal	PDGFA	97	5	18	15	0
horizontal	POLB	124	2	9	0	0
horizontal	TERT	65	14	28	26	2
horizontal	TPI1	124	1	9	1	0
horizontal	CDK11A	121	3	10	1	0
horizontal	APAF1	120	3	10	2	0
horizontal	F9	108	3	14	10	0
horizontal	ALDOC	119	0	10	6	0
horizontal	APOE	86	12	21	16	0
horizontal	VEGFA	66	24	27	16	2
horizontal	HSD17B7	116	5	11	3	0
horizontal	KLK3	124	1	8	2	0
horizontal	CASP3	121	1	9	4	0
horizontal	CYP1A2	118	5	10	2	0
horizontal	E2F1	103	11	15	6	0
horizontal	HK1	127	1	7	0	0
horizontal	ILK	124	1	8	2	0
horizontal	PGF	124	3	8	0	0
horizontal	GHRL	124	2	8	1	0
horizontal	APOA1	89	8	19	18	1

horizontal	GSS	113	8	11	2	1
horizontal	HSPA5	93	11	18	13	0
horizontal	NTRK2	120	1	9	5	0
horizontal	RRM1	129	0	6	0	0
horizontal	RFWD2	123	4	8	0	0
horizontal	ATP5B	122	3	8	2	0
horizontal	EEF1A1	110	11	12	2	0
horizontal	FOXA2	128	1	6	0	0
horizontal	LCT	125	0	7	3	0
horizontal	RARG	116	6	10	3	0
horizontal	SLC2A4	122	1	8	4	0
horizontal	SLC3A2	116	6	10	3	0
horizontal	SLC7A1	125	2	7	1	0
horizontal	TGFBR2	89	17	19	10	0
horizontal	SLC23A2	125	0	7	3	0
horizontal	TAF12	127	1	6	1	0
horizontal	KAT2B	115	6	10	4	0
horizontal	COX7A2L	124	2	7	2	0
horizontal	APC	120	5	8	2	0
horizontal	SERPINC1	117	4	9	5	0
horizontal	CASR	129	0	5	1	0
horizontal	CD40	120	7	8	0	0
horizontal	CYC1	129	1	5	0	0
horizontal	PRODH	120	3	8	4	0
horizontal	SLC34A1	123	3	7	2	0
horizontal	SLC25A1	120	4	8	3	0
horizontal	APOA5	120	2	8	5	0
horizontal	CASP2	131	0	4	0	0
horizontal	CHUK	128	2	5	0	0
horizontal	CYP2C8	119	2	8	6	0
horizontal	CYP27B1	116	3	9	7	0
horizontal	GNAS	125	4	6	0	0
horizontal	AKT2	130	1	4	0	0
horizontal	BIRC5	91	13	17	14	0
horizontal	HTR4	130	0	4	1	0
horizontal	PDX1	127	2	5	1	0
horizontal	VAMP2	127	2	5	1	0
horizontal	HNF1A	121	4	7	3	0
horizontal	TRHR	121	1	7	6	0
horizontal	ALDOA	120	2	7	6	0
horizontal	EIF2S1	132	0	3	0	0
horizontal	MC2R	132	0	3	0	0
horizontal	PLCG1	123	5	6	1	0
horizontal	NR2C1	126	2	5	2	0
horizontal	RASGRP2	126	2	5	2	0
horizontal	GLS2	108	10	11	6	0
horizontal	SLC25A5	125	4	5	1	0
horizontal	BCL2L1	102	11	12	8	2
horizontal	RPSA	128	3	4	0	0
horizontal	SLC39A1	128	1	4	2	0
horizontal	ADSL	130	1	3	1	0
horizontal	CDK2	124	3	5	3	0
horizontal	E2F4	133	0	2	0	0
horizontal	KIR2DL4	124	4	5	2	0
horizontal	XCL1	130	0	3	2	0
horizontal	UQCRB	130	1	3	1	0
horizontal	SLC16A3	124	4	5	2	0
horizontal	TLR9	121	4	6	4	0
horizontal	ABCG8	130	1	3	1	0

horizontal	CPT1B	132	1	2	0	0
horizontal	PPAT	117	6	7	5	0
horizontal	NR2C2	129	2	3	1	0
horizontal	SLC34A2	126	2	4	3	0
horizontal	TINF2	132	0	2	1	0
horizontal	ACOX1	134	0	1	0	0

The vertical-distribution type (318 genes)

assigned_type	gene_name	number of GDS				
		no-change type	correlation type	horizontal-distribution type	vertical-distribution type	no data
vertical	TAC1	76	1	1	57	0
vertical	ALDH1A1	71	6	0	58	0
vertical	NTS	55	4	13	62	1
vertical	FOS	33	30	3	69	0
vertical	CXCL1	66	10	1	58	0
vertical	CXCL10	83	1	0	51	0
vertical	CXCL11	83	1	0	51	0
vertical	SPP1	55	14	5	60	1
vertical	CXCL13	84	0	1	50	0
vertical	LPL	71	9	4	51	0
vertical	F13A1	67	15	1	52	0
vertical	MMP1	33	18	20	63	1
vertical	CCL2	60	17	4	54	0
vertical	PROS1	76	10	1	48	0
vertical	PTH	87	1	3	44	0
vertical	NR4A2	90	3	0	42	0
vertical	TNC	61	10	13	51	0
vertical	C3	87	5	1	42	0
vertical	CXCL9	93	2	0	40	0
vertical	CCL19	66	7	14	48	0
vertical	PLA2G7	90	3	2	40	0
vertical	PLAT	77	6	8	44	0
vertical	FGG	96	1	1	37	0
vertical	CYP1A1	83	4	7	41	0
vertical	OLR1	95	2	1	37	0
vertical	BDKRB1	93	4	1	37	0
vertical	MTTP	99	0	1	35	0
vertical	F3	68	10	12	45	0
vertical	IGFBP1	71	6	14	44	0
vertical	ADH1C	85	7	4	39	0
vertical	CXCL5	94	2	3	36	0
vertical	SI	64	3	22	46	0
vertical	THBS1	91	5	2	37	0
vertical	CYP1B1	90	6	2	37	0
vertical	HLA-DQB1	95	3	2	35	0
vertical	VCAM1	80	10	5	40	0
vertical	VIP	61	7	21	46	0
vertical	CCK	90	3	6	36	0
vertical	TLR2	87	9	2	37	0
vertical	DIO1	101	2	0	32	0
vertical	SLC47A1	91	8	1	35	0
vertical	CYP27A1	84	12	2	37	0
vertical	HSD3B1	81	6	10	38	0
vertical	HSD3B2	75	5	15	40	0
vertical	PDGFD	93	6	2	34	0
vertical	HLA-DPA1	94	3	5	33	0
vertical	APOB	73	6	17	39	0
vertical	COL1A2	54	20	15	45	1
vertical	KIR3DL1	79	11	8	37	0
vertical	SERPINB2	97	5	2	31	0
vertical	CETP	84	9	7	35	0
vertical	CYP4B1	96	1	7	31	0
vertical	ITGA1	105	2	0	28	0
vertical	NT5E	81	16	2	36	0

vertical	PTGS2	33	45	5	52	0
vertical	PPARG	66	12	17	40	0
vertical	NR0B2	107	1	1	26	0
vertical	SLC19A3	101	2	4	28	0
vertical	CDH1	82	13	6	34	0
vertical	HIST1H3H	109	1	0	25	0
vertical	LTC4S	105	1	3	26	0
vertical	HSPA1A	83	14	5	33	0
vertical	STAR	73	10	15	36	1
vertical	ADRB2	106	4	0	25	0
vertical	CDC6	100	7	1	27	0
vertical	ADM	111	1	0	23	0
vertical	CD36	105	1	4	25	0
vertical	GJA1	99	4	5	27	0
vertical	SERPINA1	111	0	1	23	0
vertical	SULT1E1	111	1	0	23	0
vertical	CAV1	95	11	1	28	0
vertical	NOS3	89	11	5	30	0
vertical	PF4	92	11	3	29	0
vertical	ABCB4	110	2	0	23	0
vertical	PLK1	101	7	1	26	0
vertical	TF	85	5	13	31	1
vertical	PAX6	112	0	1	22	0
vertical	PLA2G4A	103	5	2	25	0
vertical	TRH	88	6	11	30	0
vertical	COL5A2	96	8	4	27	0
vertical	FCER1G	102	8	0	25	0
vertical	CYP7B1	105	4	2	24	0
vertical	WNT4	105	3	3	24	0
vertical	F10	107	3	2	23	0
vertical	CCL11	112	0	2	21	0
vertical	HIST1H2BJ	112	1	1	21	0
vertical	AGT	90	8	9	28	0
vertical	HLA-DRB1	111	3	0	21	0
vertical	SCT	108	2	3	22	0
vertical	AKR1C4	107	5	1	22	0
vertical	EDNRB	104	5	3	23	0
vertical	G6PC	98	2	10	25	0
vertical	IBSP	59	13	23	37	3
vertical	ABCG5	110	4	0	21	0
vertical	CCR2	110	3	1	21	0
vertical	DMD	109	5	0	21	0
vertical	IGFBP6	106	2	5	22	0
vertical	AR	93	5	11	26	0
vertical	CD2	93	14	2	26	0
vertical	KDR	93	10	6	26	0
vertical	CGA	74	12	17	32	0
vertical	CYP2F1	104	3	6	22	0
vertical	GZMB	98	12	1	24	0
vertical	JUN	97	11	2	24	1
vertical	SYN1	107	5	2	21	0
vertical	ACTG2	114	3	0	18	0
vertical	APOA2	102	2	9	22	0
vertical	CCNB1	96	11	4	24	0
vertical	DIO2	105	2	7	21	0
vertical	AGTR1	119	0	0	16	0
vertical	ALB	104	4	6	21	0
vertical	HCK	110	5	1	19	0
vertical	PPBP	113	4	0	18	0

vertical	SLC1A3	104	8	2	21	0
vertical	CDK1	82	18	7	28	0
vertical	GP5	106	3	6	20	0
vertical	MAOA	100	4	9	22	0
vertical	MAOB	70	10	23	32	0
vertical	SST	112	3	2	18	0
vertical	CYP46A1	112	4	1	18	0
vertical	PREX1	115	3	0	17	0
vertical	DUSP4	111	6	0	18	0
vertical	P2RX1	111	4	2	18	0
vertical	SLC20A1	105	5	5	20	0
vertical	HTR1A	116	1	2	16	0
vertical	ITGA2	110	4	3	18	0
vertical	LHCGR	104	3	8	20	0
vertical	MGMT	116	1	2	16	0
vertical	GRAP2	95	12	5	23	0
vertical	LIPC	112	3	3	17	0
vertical	NNMT	118	1	1	15	0
vertical	PDK4	97	13	3	22	0
vertical	NR4A3	115	4	0	16	0
vertical	CD40LG	105	7	4	19	0
vertical	CYP7A1	102	2	11	20	0
vertical	CYP11A1	92	5	14	23	1
vertical	MGST1	120	1	0	14	0
vertical	POMC	96	8	9	22	0
vertical	PAK7	117	3	0	15	0
vertical	CYP17A1	98	8	8	21	0
vertical	SELP	110	7	1	17	0
vertical	CCND1	46	27	24	38	0
vertical	OXTR	115	2	3	15	0
vertical	THBD	85	16	9	25	0
vertical	CDH2	95	11	7	21	1
vertical	FN1	72	18	16	29	0
vertical	IGFBP4	102	10	4	19	0
vertical	CCL17	111	2	6	16	0
vertical	SPARC	105	10	2	18	0
vertical	APOC2	101	8	7	19	0
vertical	ASNS	110	6	3	16	0
vertical	HLA-DRA	56	29	16	34	0
vertical	LBP	119	2	1	13	0
vertical	LYN	107	7	4	17	0
vertical	PENK	80	5	24	26	0
vertical	TH	110	3	6	16	0
vertical	CXCR4	85	21	4	24	1
vertical	CD28	121	2	0	12	0
vertical	CTLA4	118	3	1	13	0
vertical	CYP2J2	90	7	15	22	1
vertical	EDN1	76	13	19	27	0
vertical	IGFBP2	115	4	2	14	0
vertical	ITGA6	103	9	5	18	0
vertical	PGR	91	6	16	22	0
vertical	PTK2	112	3	5	15	0
vertical	FMO3	96	4	15	20	0
vertical	HLA-DPB1	83	15	12	24	1
vertical	INS	105	4	9	17	0
vertical	ITGB8	84	5	22	24	0
vertical	PLTP	108	9	2	16	0
vertical	SULT2A1	105	4	9	17	0
vertical	NR1I3	117	1	4	13	0

vertical	PDGFC	99	7	10	19	0
vertical	CD34	122	2	0	11	0
vertical	CYP11B2	81	9	19	24	2
vertical	DRD1	104	3	11	17	0
vertical	F2R	95	5	15	20	0
vertical	PTHLH	104	5	9	17	0
vertical	TK1	107	6	6	16	0
vertical	TLR4	104	13	1	17	0
vertical	CCND2	106	4	9	16	0
vertical	CFTR	83	8	19	23	2
vertical	CRMP1	106	9	4	16	0
vertical	FES	103	13	2	17	0
vertical	L1CAM	121	3	0	11	0
vertical	VIPR1	121	2	1	11	0
vertical	CYP2C19	117	6	0	12	0
vertical	NR5A2	123	1	1	10	0
vertical	SMAD7	117	6	0	12	0
vertical	CX3CL1	111	4	6	14	0
vertical	ASS1	125	1	0	9	0
vertical	ATP1B1	98	11	8	18	0
vertical	AVP	119	4	1	11	0
vertical	CCR5	101	8	9	17	0
vertical	CYP2E1	116	4	3	12	0
vertical	F8	119	2	3	11	0
vertical	HGF	110	6	5	14	0
vertical	MC4R	116	4	3	12	0
vertical	MYL3	125	1	0	9	0
vertical	PRKCB	119	5	0	11	0
vertical	TNNI1	125	0	1	9	0
vertical	CYP21A2	118	3	3	11	0
vertical	HSD11B1	106	9	5	15	0
vertical	ITGAX	93	13	9	19	1
vertical	SCTR	109	9	3	14	0
vertical	CLDN2	121	2	2	10	0
vertical	TLR7	121	4	0	10	0
vertical	CCNA2	75	24	11	25	0
vertical	FGA	108	2	11	14	0
vertical	HES1	123	2	1	9	0
vertical	PTH1R	120	2	3	10	0
vertical	TGFB2	99	11	8	17	0
vertical	XDH	111	4	7	13	0
vertical	IRS2	99	13	6	17	0
vertical	SPHK1	123	2	1	9	0
vertical	SLCO1B3	123	3	0	9	0
vertical	SLC15A1	119	4	2	10	0
vertical	TLR3	98	13	7	17	0
vertical	TM7SF2	122	4	0	9	0
vertical	NRP1	125	2	0	8	0
vertical	ABCA1	124	3	0	8	0
vertical	CD151	127	1	0	7	0
vertical	CYP3A7	97	7	14	17	0
vertical	FGB	118	2	5	10	0
vertical	GAS6	118	5	2	10	0
vertical	KISS1	115	6	3	11	0
vertical	CYP4F3	121	4	1	9	0
vertical	NPY	106	5	10	14	0
vertical	SLC2A2	127	1	0	7	0
vertical	TNF	70	17	20	25	3
vertical	TXN	112	7	4	12	0

vertical	UGT2B15	124	2	1	8	0
vertical	ABCC3	115	4	5	11	0
vertical	CD247	123	4	0	8	0
vertical	DFFB	126	1	1	7	0
vertical	FABP6	117	2	6	10	0
vertical	SLC1A2	126	1	1	7	0
vertical	SLCO1B1	129	0	0	6	0
vertical	CD226	126	1	1	7	0
vertical	CYSLTR1	126	1	1	7	0
vertical	APOA4	110	6	7	12	0
vertical	NFKBIA	125	3	0	7	0
vertical	PECAM1	122	5	0	8	0
vertical	UGDH	119	4	3	9	0
vertical	KLF4	125	2	1	7	0
vertical	MLXIPL	122	4	1	8	0
vertical	ENO3	124	1	3	7	0
vertical	ITGA4	124	0	4	7	0
vertical	ITGAD	106	4	12	13	0
vertical	SRC	121	3	3	8	0
vertical	UGT2B7	127	2	0	6	0
vertical	NR1H4	130	0	0	5	0
vertical	PLCB1	127	2	0	6	0
vertical	ULBP1	109	3	11	12	0
vertical	CDK6	126	1	2	6	0
vertical	COL4A1	123	2	3	7	0
vertical	NQO1	81	17	16	21	0
vertical	F12	129	0	1	5	0
vertical	IDH1	120	6	1	8	0
vertical	ITGA2B	108	8	7	12	0
vertical	ITGB3	114	5	6	10	0
vertical	JUP	120	3	4	8	0
vertical	GP6	116	1	8	9	1
vertical	ANGPT1	122	4	2	7	0
vertical	COL4A2	125	3	1	6	0
vertical	DDC	125	0	4	6	0
vertical	FGF1	125	4	0	6	0
vertical	GK	128	2	0	5	0
vertical	GRPR	95	15	9	16	0
vertical	IGFBP3	122	3	3	7	0
vertical	TIMP1	113	9	3	10	0
vertical	ABCA7	125	4	0	6	0
vertical	APOBEC3G	122	3	3	7	0
vertical	JAG1	118	6	3	8	0
vertical	APOC3	97	9	14	15	0
vertical	FCGR3A	130	1	0	4	0
vertical	PRKCA	124	4	1	6	0
vertical	SSTR2	112	6	7	10	0
vertical	ABCB11	124	2	3	6	0
vertical	SLC19A2	112	9	4	10	0
vertical	ITGA11	127	2	1	5	0
vertical	CD244	118	4	5	8	0
vertical	CDC20	126	4	0	5	0
vertical	F11	126	4	0	5	0
vertical	PFKM	120	2	6	7	0
vertical	WARS2	123	3	3	6	0
vertical	TREH	129	2	0	4	0
vertical	BRCA2	95	13	12	15	0
vertical	CD3G	125	4	1	5	0
vertical	CDC25C	116	7	4	8	0

vertical	COL4A4	131	1	0	3	0
vertical	CYP11B1	125	4	1	5	0
vertical	GSN	125	4	1	5	0
vertical	MYH6	122	2	5	6	0
vertical	MYOG	113	8	5	9	0
vertical	CCL3	128	3	0	4	0
vertical	SKP2	128	2	1	4	0
vertical	SOX2	125	2	3	5	0
vertical	HIST2H4A	131	1	0	3	0
vertical	CHEK2	122	5	2	6	0
vertical	BAK1	124	4	2	5	0
vertical	HK2	133	0	0	2	0
vertical	APLN	127	3	1	4	0
vertical	NR1D1	133	0	0	2	0
vertical	RPS6KA6	130	2	0	3	0
vertical	NEUROG3	133	0	0	2	0
vertical	ADRB3	129	1	2	3	0
vertical	CYP8B1	132	1	0	2	0
vertical	HLA-B	129	2	1	3	0
vertical	INSR	117	6	5	7	0
vertical	SLC10A1	126	3	2	4	0
vertical	NPC1L1	132	1	0	2	0
vertical	ALOX5	107	9	9	10	0
vertical	CD47	134	0	0	1	0
vertical	GP9	122	4	4	5	0
vertical	SLC5A5	122	4	4	5	0
vertical	AMACR	131	1	1	2	0
vertical	HIST2H4B	134	0	0	1	0

Unclassified genes (40 genes)

assigned_type	gene_name	number of GDS				
		no-change type	correlation type	horizontal-distribution type	vertical-distribution type	no data
un-classified	IKBKE	135	0	0	0	0
un-classified	NTRK1	133	0	1	1	0
un-classified	UGT1A8	133	0	1	1	0
un-classified	GJD2	133	0	1	1	0
un-classified	SLC46A1	133	1	0	1	0
un-classified	KLRC1	131	2	2	0	0
un-classified	ITGA10	131	0	2	2	0
un-classified	TLR10	131	0	2	2	0
un-classified	NPM1	130	2	1	2	0
un-classified	AURKA	130	2	1	2	0
un-classified	ACACB	129	2	2	2	0
un-classified	BAAT	129	3	3	0	0
un-classified	FUS	129	2	2	2	0
un-classified	PTPN1	129	3	3	0	0
un-classified	PSENEN	129	3	0	3	0
un-classified	ACLY	128	3	3	1	0
un-classified	LIPE	126	1	4	4	0
un-classified	IRF7	125	5	0	5	0
un-classified	TEK	124	4	4	3	0
un-classified	BCL2L11	124	4	3	4	0
un-classified	PTCH1	123	4	4	4	0
un-classified	HMGA1	122	5	3	5	0
un-classified	HRAS	122	6	6	1	0
un-classified	DAB2	121	5	4	5	0
un-classified	DNASE2	121	6	6	2	0
un-classified	F2	119	7	2	7	0
un-classified	LCAT	119	2	7	7	0
un-classified	PTEN	119	8	8	0	0
un-classified	IGF2R	117	7	4	7	0
un-classified	CYP51A1	116	7	7	5	0
un-classified	HSP90AB1	114	9	9	3	0
un-classified	EIF4E	112	10	10	3	0
un-classified	ITGA5	109	10	10	6	0
un-classified	ADA	107	10	8	10	0
un-classified	TNFSF10	107	13	2	13	0
un-classified	DBF4	106	11	7	11	0
un-classified	CYB5A	101	13	13	7	1
un-classified	MET	96	11	14	14	0
un-classified	RB1	95	16	7	16	1
un-classified	COL1A1	89	20	5	20	1
