# Supplementary material for: Relationship between regulatory pattern of gene expression level and gene function
Source: PLoS One. 2017 May 11;12(5):e0177430. doi: 10.1371/journal.pone.0177430 (PMC5426767; doi:10.1371/journal.pone.0177430)
Supplement: S3 Table — The category (hierarchical framework), the pathway name, and the name of involved genes are shown for each selected pathway. (PDF) [file pone.0177430.s003.pdf]

S3 Table : List of the selected pathways from pathway analysis

Category	Pathway name	Gene									
Correlation type											
Cell Cycle	REACTOME_P53_INDEPENDENT_G1_S_DNA_DAMAGE_CHECKPOINT	CHEK1	PSMB9	PSMB10	CDC25A						
Cell Cycle	REACTOME_G1_S_TRANSITION	CDKN1A	CDKN1B	PCNA	POLA1	PSMB9	PSMB10	TYMS	CCNE1	CDC25A	
Cell Cycle	REACTOME_G1_S_SPECIFIC_TRANSCRIPTION	PCNA	POLA1	TYMS	CCNE1	CDC25A					
Cell Cycle	REACTOME_S_PHASE	CDKN1A	CDKN1B	PCNA	POLA1	POLD1	PSMB9	PSMB10	CCNE1	CDC25A	
DNA Repair	REACTOME_NUCLEOTIDE_EXCISION_REPAIR	DDB2	PCNA	POLD1							
DNA Repair	REACTOME_GLOBAL_GENOMIC_NER_GG_NER	DDB2	PCNA	POLD1							
DNA Replication	REACTOME_PROCESSIVE_SYNTHESIS_ON_THE_LAGGING_STRAND	PCNA	POLA1	POLD1							
DNA Replication	REACTOME_POL_SWITCHING	PCNA	POLA1	POLD1							
DNA Replication	REACTOME_SYNTHESIS_OF_DNA	CDKN1A	CDKN1B	PCNA	POLA1	POLD1	PSMB9	PSMB10			
DNA Replication	REACTOME_LAGGING_STRAND_SYNTHESIS	PCNA	POLA1	POLD1							
DNA Replication	REACTOME_DNA_REPLICATION	CDKN1A	CDKN1B	PCNA	POLA1	POLD1	PSMB9	PSMB10	AURKB		
DNA Replication	REACTOME_DNA_STRAND_ELONGATION	PCNA	POLA1	POLD1							
Immune System	REACTOME_IMMUNE_SYSTEM	CDKN1A	CDKN1B	TICAM1	CTSL	AKT1	LY96	LAT	CD274	HLA-G	ICAM1
		ITGAV	ITGB2	ITGB7	LCK	MYD88	NCAM1	NFKB2	PIK3CA	PSMB9	PSMB10
		PTPN6	STAT1	WAS	CASP8	IFITM1	CD3E	CD8A	CD14	CD19	CD86
Immune System	REACTOME_ADAPTIVE_IMMUNE_SYSTEM	CDKN1A	CDKN1B	CTSL	AKT1	LAT	CD274	HLA-G	ICAM1	ITGAV	ITGB2
		ITGB7	LCK	PIK3CA	PSMB9	PSMB10	PTPN6	WAS	IFITM1	CD3E	CD8A
		CD19	CD86								
Programmed Cell Death	REACTOME_APOPTOSIS	AKT1	BBC3	FAS	PMAIP1	PSMB9	PSMB10	BAX	BID	VIM	CASP8
		TNFRSF10B									
Metabolism(K)	KEGG_PYRIMIDINE_METABOLISM	TYMS	POLA1	POLD1	CAD	TYMP					
Environmental Information Processing	KEGG_CELL_ADHESION_MOLECULES_CAMS	CD86	CD274	ITGAM	HLA-G	ITGB7	ITGAV	SPN	ITGB2	CD8A	HLA-E
		ICAM1	NCAM1	CLDN7							
Cellular Processes	KEGG_P53_SIGNALING_PATHWAY	CASP8	IGF1	BID	BAX	PMAIP1	CDKN1A	BBC3	TNFRSF10B	DDB2	CHEK1
		SERPINE1	FAS	CCNE1							
Organismal Systems	KEGG_TOLL_LIKE_RECEPTOR_SIGNALING_PATHWAY	CD86	STAT1	MYD88	CCL5	CCL4	LY96	AKT1	CD14	CASP8	TICAM1
		CXCL8	PIK3CA								
Organismal Systems	KEGG_NATURAL_KILLER_CELL_MEDIATED_CYTOTOXICITY	HLA-G	ITGB2	HLA-E	LCK	ICAM1	PTPN6	BID	LAT	TNFRSF10B	PIK3CA
		FAS									
Pathways of replication, repair, gene expression, protein biosynthesis	PID_E2F_PATHWAY	POLA1	PLAU	CCNE1	CDC25A	CDKN1A	BRCA1	SERPINE1	CDKN1B	TYMS	
Transcription factor mediated signaling pathways<br>Cytokine and chemokine mediated signaling pathways	PID_MYC_REPRESSPATHWAY	SLC11A1	CCL5	FTH1	DDIT3	CDKN1A	CDKN1B	BRCA1			
Glycoconjugated protein signaling pathway	PID_AMB2_NEUTROPHILS_PATHWAY	ITGB2	AKT1	LCK	PLAU	TLN1	ITGAM	PLAUR	ICAM1		
Signaling pathways pertinent to development	PID_INTEGRIN5_PATHWAY	ITGAV	TGFBR1	ITGB7	PLAU	PLAUR					
Apoptosis<br>Cell Signaling	BIOCARTA_BAD_PATHWAY	IGF1	BAX	AKT1	PIK3CA						
Horizontal distribution type											
Metabolism(R)	REACTOME_GLUCONEOGENESIS	ALDOA	ALDOC	GAPDH	SLC25A1	TPI1					
Metabolism(R)	REACTOME_RESPIRATORY_ELECTRON_TRANSPORT	CYC1	SDHA	UQCRB	COX7A2L						
Metabolism(R)	REACTOME_RESPIRATORY_ELECTRON_TRANSPORT_ATP_SYNTHESIS_BY_CHEMIOSMOTIC_COUPLING_AND_HEAT_PRODUCTION_BY_UNCOUPLING_PROTEINS_	CYC1	ATP5B	SDHA	UQCRB	COX7A2L					
Signal Transduction	REACTOME_SIGNALING_BY_WNT	CSNK1A1	CTNNB1	APC	PPP2CA	PSMD10	SKP1				
Signal Transduction	REACTOME_CTNNB1_PHOSPHORYLATION_CASCADE	CSNK1A1	CTNNB1	APC	PPP2CA						
Metabolism(K)	KEGG_OXIDATIVE_PHOSPHORYLATION	COX7A2L	SDHA	ATP5B	UQCRB	CYC1					
Human Diseases	KEGG_ALZHEIMERS_DISEASE	APOE	qAPAF1	APH1A	GAPDH	COX7A2L	CDK5R1	GNAQ	SDHA	ATP5B	CASP3
		UQCRB	PSEN1	APP	CYC1						
Human Diseases	KEGG_PARKINSONS_DISEASE	qAPAF1	COX7A2L	SLC25A5	SDHA	ATP5B	CASP3	UQCRB	CYC1	SLC6A3	
Human Diseases	KEGG_HUNTINGTONS_DISEASE	CREB1	qAPAF1	COX7A2L	GNAQ	SLC25A5	SDHA	ATP5B	SOD1	CASP3	UQCRB
		TP53	CYC1								
Human Diseases	KEGG_PANCREATIC_CANCER	E2F1	CHUK	PGF	ERBB2	AKT2	NFKB1	EGFR	RELA	VEGFA	TGFBR2
		BCL2L1	CDK4	TP53							
Human Diseases	KEGG_PROSTATE_CANCER	E2F1	CHUK	GSTP1	ERBB2	FGFR2	MDM2	CREB1	AKT2	NFKB1	EGFR
		RELA	CTNNB1	HSP90AA1	BCL2	PDGFA	CDK2	TP53	KLK3		
Cytokine and chemokine mediated signaling pathways	PID_IL2_PI3KPATHWAY	NFKB1	RELA	BCL2	BCL2L1	HSP90AA1	E2F1	TERT			
Growth factor signaling pathways	PID_BETACATENIN_DEG_PATHWAY	SKP1	APC	CTNNB1	CSNK1A1						
Hormone signaling pathways	PID_AR_TF_PATHWAY	KAT2B	NR2C2	MDM2	HSP90AA1	NR3C1	NR2C1	KLK3			
Adhesion<br>Apoptosis	BIOCARTA_AKT_PATHWAY	CHUK	RELA	GH1	PPP2CA	FASLG	HSP90AA1	NFKB1			
Cell Signaling	BIOCARTA_PS1_PATHWAY	APC	PSEN1	CTNNB1	HNF1A						
Expression	BIOCARTA_RNA_PATHWAY	CHUK	RELA	EIF2S1	NFKB1	TP53					
Neuroscience	BIOCARTA_P35ALZHEIMERS_PATHWAY	CDK5R1	PPP2CA	CSNK1A1	APP						
	ST_WNT_BETA_CATENIN_PATHWAY	PSEN1	RPSA	TSHB	APC	CTNNB1	AKT2	CSNK1A1			

Category	Pathway name	Gene									
Vertical distribution type											
Metabolism(R)	REACTOME_BILE_ACID_AND_BILE_SALT_METABOLISM	SLCO1B1	CYP46A1	AKR1C4	CYP7A1	CYP8B1	CYP27A1	ALB	FABP6	AMACR	SLCO1B3
		SLC10A1	ABCB11	ABCC3	CYP7B1						
Metabolism(R)	REACTOME_METABOLISM_OF_STEROID_HORMONES_AND_VITAMINS_A_AND_D	CGA	CYP11A1	CYP11B1	CYP11B2	CYP17A1	CYP21A2	HSD3B1	HSD3B2	HSD11B1	POMC
		STAR									
Metabolism(R)	REACTOME_PHASE1_FUNCTIONALIZATION_OF_COMPOUNDS	CYP46A1	ADH1C	CYP1A1	CYP1B1	CYP3A7	CYP2C19	CYP2E1	CYP2F1	CYP2J2	CYP4B1
		CYP7A1	CYP8B1	CYP11A1	CYP11B1	CYP11B2	CYP17A1	CYP21A2	CYP27A1	ALDH1A1	FMO3
		CYP4F3	MAOA	MAOB	POMC	PTGS2	CYP7B1				
Metabolism(R)	REACTOME_ENDOGENOUS_STEROLS	CYP46A1	CYP1B1	CYP7A1	CYP8B1	CYP11A1	CYP11B1	CYP11B2	CYP17A1	CYP21A2	CYP27A1
		POMC	CYP7B1								
Metabolism(R)	REACTOME_METABOLISM_OF_LIPIDS_AND_LIPOPROTEINS	SLCO1B1	CETP	CGA	CYP46A1	AKR1C4	CYP1A1	CYP7A1	CYP8B1	CYP11A1	CYP11B1
		CYP11B2	CYP17A1	CYP21A2	CYP27A1	AGT	ABCA1	ALB	FABP6	AMACR	GK
		SLCO1B3	HSD3B1	HSD3B2	HSD11B1	APOA2	APOB	IDH1	APOC2	APOC3	LIPC
		LPL	MTTP	ABCB4	PLA2G4A	PLTP	POMC	PPARG	ABCG5	SLC10A1	STAR
		SULT2A1	TM7SF2	CAV1	ABCB11	ABCC3	SPHK1	CYP7B1	CD36	NR1D1	
Signal Transduction	REACTOME_SIGNALING_BY_GPCR	CXCL13	CYSLTR1	CGA	CCR2	CCR5	ADM	ADRB2	ADRB3	DRD1	AGT
		AGTR1	EDN1	EDNRB	F2R	PLCB1	CXCL1	GRPR	HTR1A	CXCL10	KISS1
		LHCGR	MC4R	CXCL9	NPY	NTS	OXTR	PENK	PF4	PLA2G4A	WNT4
		POMC	PPBP	AVP	PRKCA	PRKCB	PTH	PTHLH	PTH1R	PREX1	BDKRB1
		SCT	SCTR	CCL2	CCL3	CCL11	CCL17	CCL19	CXCL11	CXCL5	CX3CL1
		SST	SSTR2	TAC1	C3	TRH	VIP	VIPR1	CXCR4	CCK	CDK1
Signal Transduction	REACTOME_PEPTIDE_LIGAND_BINDING_RECEPTORS	CXCL13	CCR2	CCR5	AGT	AGTR1	EDN1	EDNRB	F2R	CXCL1	GRPR
		CXCL10	KISS1	MC4R	CXCL9	NPY	NTS	OXTR	PENK	PF4	POMC
		PPBP	AVP	BDKRB1	CCL2	CCL3	CCL11	CCL17	CCL19	CXCL11	CXCL5
		CX3CL1	SST	SSTR2	TAC1	C3	TRH	CXCR4	CCK		
Signal Transduction	REACTOME_CLASS_A1_RHODOPSIN_LIKE_RECEPTORS	CXCL13	CYSLTR1	CGA	CCR2	CCR5	ADRB2	ADRB3	DRD1	AGT	AGTR1
		EDN1	EDNRB	F2R	CXCL1	GRPR	HTR1A	CXCL10	KISS1	LHCGR	MC4R
		CXCL9	NPY	NTS	OXTR	PENK	PF4	POMC	PPBP	AVP	BDKRB1
		CCL2	CCL3	CCL11	CCL17	CCL19	CXCL11	CXCL5	CX3CL1	SST	SSTR2
		TAC1	C3	TRH	CXCR4	CCK					
Signal Transduction	REACTOME_GPCR_DOWNSTREAM_SIGNALING	CXCL13	CYSLTR1	CGA	CCR2	CCR5	ADM	ADRB2	ADRB3	DRD1	AGT
		AGTR1	EDN1	EDNRB	F2R	PLCB1	CXCL1	GRPR	HTR1A	CXCL10	KISS1
		LHCGR	MC4R	CXCL9	NPY	NTS	OXTR	PENK	PF4	POMC	PPBP
		AVP	PRKCA	PRKCB	PTH	PTHLH	PTH1R	PREX1	BDKRB1	SCT	SCTR
		CCL19	CXCL11	CXCL5	SST	SSTR2	TAC1	C3	TRH	VIP	VIPR1
		CXCR4	CCK								
Signal Transduction	REACTOME_GPCR_LIGAND_BINDING	CXCL13	CYSLTR1	CGA	CCR2	CCR5	ADM	ADRB2	ADRB3	DRD1	AGT
		AGTR1	EDN1	EDNRB	F2R	CXCL1	GRPR	HTR1A	CXCL10	KISS1	LHCGR
		MC4R	CXCL9	NPY	NTS	OXTR	PENK	PF4	WNT4	POMC	PPBP
		AVP	PTH	PTHLH	PTH1R	BDKRB1	SCT	SCTR	CCL2	CCL3	CCL11
		CCL17	CCL19	CXCL11	CXCL5	CX3CL1	SST	SSTR2	TAC1	C3	TRH
		VIP	VIPR1	CXCR4	CCK						
	REACTOME_STEROID_HORMONES	CGA	CYP11A1	CYP11B1	CYP11B2	CYP17A1	CYP21A2	HSD3B1	HSD3B2	HSD11B1	POMC
		STAR									
Metabolism(K)	KEGG_STEROID_HORMONE_BIOSYNTHESIS	AKR1C4	HSD3B2	HSD3B1	CYP7A1	CYP11A1	SULT1E1	HSD11B1	CYP21A2	CYP17A1	CYP11B2
		CYP11B1	CYP3A7	CYP1A1	CYP1B1	CYP7B1	UGT2B7	UGT2B15			
Human Diseases	KEGG_ASTHMA	TNF	CCL11	FCER1G	CD40LG	HLA-DPA1	HLA-DPB1	HLA-DQB1	HLA-DRB1	HLA-DRA	
Human Diseases	KEGG_SYSTEMIC_LUPUS_ERYTHEMATOSUS	CD28	FCGR3A	HIST2H4B	HLA-DPA1	HLA-DPB1	HLA-DQB1	HIST2H4A	C3	HLA-DRB1	HLA-DRA
		TNF	HIST1H2BJ	HIST1H3H	CD40LG						
